# Leading causes of death in Tunisia, 2020: Salsabil Rejaibi

**DOI:** 10.1093/eurpub/ckac131.218

**Published:** 2022-10-25

**Authors:** S Rejaibi, N Zoghalami, A Silini, I Ben Slama, A Skhiri, I Mariem, I Mensi, MA Bennour, S Taibi, H Aounallah-Skhiri

**Affiliations:** 1 Epidemiology Department, NIH Tunisia, Tunis, Tunisia; 2 Medical Faculty of Medicine, Tunis El Manar University, Tunis, Tunisia; 3 Nutrition Surveillance and Epidemiology, SURVEN, Research Laboratory, Tunis, Tunisia; 4 Epidemiology and Statistics Department, Abderrahman Mami Hospital, Tunis, Tunisia; 5 UNFPA, Tunis, Tunisia

## Abstract

**Background:**

Mortality data represent a primary source of information for monitoring a population health status over years. In Tunisia, the national Information System on Causes of Death (ISCD) lacks completeness (average coverage rate of 40%); however, in order to examine covid-19’s effect on mortality data, the ISCD was reinforced. We aimed to give an overview of leading causes of death in Tunisia for 2020.

**Methods:**

Data were obtained from Medical Certificates of Cause Of Death (MCCOD) sent by municipalities to the National Institute of Health in accordance with the legislative framework. Causes of Death (CoD) coding process was performed based on the International Classification of Diseases, Tenth Revision (ICD-10). The underlying cause of death was identified based on IRIS software, and mortality statistics were presented based on the world health organization cause-of-death lists for tabulating mortality statistics. Data analysis was performed using SPSS software.

**Results:**

A total of 46.420 MCCOD among 75.365 deaths officially declared by the National Institute of Statistics, were analyzed (coverage rate of 61.2%). The 10 leading causes of death for both sexes, in rank order were: diabetes mellitus, cerebrovascular diseases, covid-19, ischemic heart diseases, external causes of death, digestive and pulmonary malignant neoplasms, conditions of neonatal period, hypertensive diseases, and influenza and pneumonia. Leading causes of infant deaths were: certain conditions originating in perinatal period, congenital malformations, deformations and chromosomal abnormalities, diseases of respiratory system, certain infectious and parasitic diseases, and diseases of nervous system.

**Conclusions:**

The COVID-19 pandemic was an opportunity to improve the Tunisian ISCD’s coverage rate. However, efforts should be maintained to optimize system completeness, and decision makers should be more sensitized regarding the urgent need for system digitalization.

**Key messages:**

• Mortality statistics have shown that covid-19 ranks third among leading causes of death in Tunisia for 2020; and non communicable disease accounted for 6 out of 10 leading causes of deaths.

• The ISCD coverage rate was improved in 2020 reaching 61.2%; however the system digitalization is an essential and sustainable solution to optimize completeness.

